# Researching Complex Interventions in Health: The State of the Art: Exeter, UK. 14-15 October 2015

**DOI:** 10.1186/s12913-016-1274-0

**Published:** 2016-04-04

**Authors:** Peter Craig, Ingalill Rahm-Hallberg, Nicky Britten, Gunilla Borglin, Gabriele Meyer, Sascha Köpke, Jane Noyes, Jackie Chandler, Sara Levati, Anne Sales, Lehana Thabane, Lora Giangregorio, Nancy Feeley, Sylvie Cossette, Rod Taylor, Jacqueline Hill, David A. Richards, Willem Kuyken, Louise von Essen, Andrew Williams, Karla Hemming, Richard Lilford, Alan Girling, Monica Taljaard, Munyaradzi Dimairo, Mark Petticrew, Janis Baird, Graham Moore, Willem Odendaal, Salla Atkins, Elizabeth Lutge, Natalie Leon, Simon Lewin, Katherine Payne, Theo van Achterberg, Walter Sermeus, Martin Pitt, Thomas Monks

**Affiliations:** MRC/CSO Social and Public Health Sciences Unit (SPHSU), University of Glasgow, 200 Renfield Street, Glasgow, G2 3QB UK; Lund University, PO Box 117, 221 00 Lund, Sweden; University of Exeter Medical School, South Cloisters, St Luke’s Campus, Exeter, EX1 2LU UK; Department of Caring Science, Malmö University, SE-205 06 Malmö, Sweden; Martin Luther University Halle-Wittenberg, Magdeburger Str. 8, D-06112 Halle (Saale), Germany; University of Lübeck, ᅟ, Germany; Bangor University, ᅟ, UK; Cochrane Collaboration, Oxford, UK; NMAHP Research Unit, Glasgow Caledonian University, ᅟ, UK; School of Nursing, University of Michigan, USA and Center for Clinical Management Research, VA Ann Arbor Healthcare System, ᅟ, USA; McMaster University, ᅟ, Canada; University of Waterloo, ᅟ, Canada; Ingram School of Nursing, McGill University, and Centre for Nursing Research and Lady Davis Institute, Jewish General Hospital, Montreal, Québec Canada; Faculty of Nursing, University of Montreal, and Montreal Heart Institute Research Center, Montreal, Québec Canada; University of Exeter Medical School, South Cloisters, St Luke’s Campus, ᅟ, UK; Mood Disorders Centre, School of Psychology, University of Exeter, ᅟ, UK; University of Exeter Medical School, ᅟ, UK; Department of Psychiatry, University of Oxford, ᅟ, UK; Department of Public Health and Caring Sciences, Uppsala University, ᅟ, Sweden; Farr Institute, Scotland, and the Scottish Collaboration for Public Health Research and Policy, 20 West Richmond Street (East Wing), Edinburgh, EH8 9DX UK; University of Birmingham Medical School, ᅟ, UK; Warwick Medical School, University of Warwick, Coventry, UK; Institute of Applied Health Research, College of Medical and Dental Sciences, University of Birmingham, ᅟ, UK; Clinical Epidemiology Program, Ottawa Hospital Research Institute, Ottawa, Canada; University of Sheffield, ᅟ, UK; Faculty of Public Health and Policy, London School of Hygiene & Tropical Medicine, ᅟ, UK; MRC Lifecourse Epidemiology Unit, University of Southampton, ᅟ, UK; DECIPHer, Cardiff University, ᅟ, UK; Health Systems Research Unit, South African Medical Research Council, South Africa, http://www.mrc.ac.za/healthsystems/healthsystems.htm; Karolinska Institutet, Stockholm, Sweden; Epidemiology, Health Research and Knowledge Management, KwaZulu-Natal Department of Health, ᅟ, South Africa; South African Medical Research Council, ᅟ, South Africa; Global Health Unit, Norwegian Knowledge Centre for the Health Services, ᅟ, Norway; The University of Manchester, ᅟ, UK; Leuven University KU Leuven, ᅟ, Belgium; School of Public Health & Primary Care, Leuven University KU Leuven, ᅟ, Belgium; University of Exeter Medical School, ᅟ, UK; Faculty of Health Sciences, University of Southampton, ᅟ, UK

## Abstract

KEYNOTE PRESENTATIONS

K1 Researching complex interventions: the need for robust approaches

Peter Craig

K2 Complex intervention studies: an important step in developing knowledge for practice

Ingalill Rahm-Hallberg

K3 Public and patient involvement in research: what, why and how?

Nicky Britten

K4 Mixed methods in health service research – where do we go from here?

Gunilla Borglin

SPEAKER PRESENTATIONS

S1 Exploring complexity in systematic reviews of complex interventions

Gabriele Meyer, Sascha Köpke, Jane Noyes, Jackie Chandler

S2 Can complex health interventions be optimised before moving to a definitive RCT? Strategies and methods currently in use

Sara Levati

S3 A systematic approach to develop theory based implementation interventions

Anne Sales

S4 Pilot studies and feasibility studies for complex interventions: an introduction

Lehana Thabane, Lora Giangregorio

S5 What can be done to pilot complex interventions?

Nancy Feeley, Sylvie Cossette

S6 Using feasibility and pilot trials to test alternative methodologies and methodological procedures prior to full scale trials

Rod Taylor

S7 A mixed methods feasibility study in practice

Jacqueline Hill, David A Richards, Willem Kuyken

S8 Non-standard experimental designs and preference designs

Louise von Essen

S9 Evaluation gone wild: using natural experimental approaches to evaluate complex interventions

Andrew Williams

S10 The stepped wedge cluster randomised trial: an opportunity to increase the quality of evaluations of service delivery and public policy interventions

Karla Hemming, Richard Lilford, Alan Girling, Monica Taljaard

S11 Adaptive designs in confirmatory clinical trials: opportunities in investigating complex interventions

Munyaradzi Dimairo

S12 Processes, contexts and outcomes in complex interventions, and the implications for evaluation

Mark Petticrew

S13 Processes, contexts and outcomes in complex interventions, and the implications for evaluation

Janis Baird, Graham Moore

S14 Qualitative evaluation alongside RCTs: what to consider to get relevant and valuable results

Willem Odendaal, Salla Atkins, Elizabeth Lutge, Natalie Leon, Simon Lewin

S15 Using economic evaluations to understand the value of complex interventions: when maximising health status is not sufficient

Katherine Payne

S16 How to arrive at an implementation plan

Theo van Achterberg

S17 Modelling process and outcomes in complex interventions

Walter Sermeus

S18 Systems modelling for improving health care

Martin Pitt, Thomas Monks

## KEYNOTE PRESENTATIONS

### K1 Researching complex interventions: the need for robust approaches

#### Peter Craig (peter.craig@glasgow.ac.uk)

##### MRC/CSO Social and Public Health Sciences Unit (SPHSU), University of Glasgow, 200 Renfield Street, Glasgow G2 3QB, UK

Since the revised MRC guidance on the development and evaluation of complex interventions was published in 2008, much progress has been made in developing and defining good practice, and there is an increasingly detailed and comprehensive set of guidance available to help researchers and funders make the right choices. But progress is patchy, and evidence continues to accumulate of waste in research. Robust approaches are needed, that combine good practice across all stages of the evaluation process, from the initial choice and framing of research questions, through to implementation and translation of evidence. The elements of a robust approach are mostly well-known, but much more rarely are they combined into a coherent package. The talk will consider why this is so, and what can be done to improve matters.

### K2 Complex intervention studies: an important step in developing knowledge for practice

#### Ingalill Rahm-Hallberg (ingalill.rahm_hallberg@rektor.lu.se)

##### Lund University, PO Box 117, 221 00 Lund, Sweden

This presentation will provide the history of the “Complex Interventions in Health” book. The development of the book took off in the European Academy of Nursing Science (EANS) summer school. For more than 12 years the EANS has gathered doctoral students from all over Europe in a three year programme. The content developed over time, inspired by the critique stating that nursing research was heavily dominated by descriptive, cross-sectional or qualitative research not really informing practice. This lead to building a curriculum based on the MRC guidance on complex interventions and the teaching programme became truly successful among students. The road to developing the content for a book was short. Professor Richards and I played around on the black board and after that it has been a success-story. So many authors delivering on time! However, rightfully we have been told that research in health is more than researching complex interventions. I cannot agree more, it is only one step, but a very important one. Health research, as any research with aspirations for informing practice, needs to be carried out systematically and programmatically, using a variety of designs and methods. It is helpful to think of knowledge development as being a stepwise process starting off with discovery and once possible going into the phase of evaluation and once solid knowledge is obtained it is about implementation in practice.

### K3 Public and patient involvement in research: what, why and how?

#### Nicky Britten (N.Britten@exeter.ac.uk)

##### University of Exeter Medical School, South Cloisters, St Luke’s Campus, Exeter EX1 2LU, UK

In this presentation I will explain what PPI is, what it isn’t, and how it differs from engagement, as there is some misunderstanding. There is a range of both ethical and pragmatic reasons for involving members of the public and patients in research, depending on the different values and perspectives of those involved. I will provide practical examples of PPI at different stages of the research cycle: identifying and prioritising research questions; developing research proposals; doing research; analysing research data; disseminating and implementing findings; as well as PPI in systematic reviewing and operational research. Drawing on my own research, I will use the particular example of the Diabetes Intervention for Agenda Trial (DIAT) to illustrate PPI in a trial from start to finish. I will present a theoretical framework for characterising PPI which paradoxically is of practical value in reflecting on PPI practice. Finally I will present the Public Involvement Impact Assessment Framework (the PiiAF) for helping to evaluate the various intended and unintended impacts of PPI.

### K4 Mixed methods in health service research – where do we go from here?

#### Gunilla Borglin (gunilla.borglin@mah.se)

##### Department of Caring Science, Malmö University, SE-205 06 Malmö, Sweden

In the 21st century, it is more obvious than ever before that health services research and its practitioners exist in an extremely complex contextual environment. Consequently, at the heart of understanding how to deliver an evidence base for safe and effective care in a setting characterised by multifaceted health care demands, is the realisation that no one research method in isolation will suffice. This realisation, together with the REWARD group’s contention that 85 % of health service research conducted – and most importantly mainly funded by public money – is ‘waste’, is one of the reasons why frameworks such as the MRC guidance on complex interventions, and mixed methods designs are subject to intensified attention. This plenary address aims to discuss the contribution of mixed methods to researching complex interventions within the MRC framework and to consider its place in contemporary health service research. The MRC framework for researching complex interventions has highlighted the importance of research designs including both qualitative and quantitative approaches. However when reviewing the literature prior to writing my chapter in our recent textbook, it was clear that designing and conducting truly mixed methods research presents health service researchers with a number of challenges. Predominant amongst these are the selection of designs from suggested typologies, methodological reporting, critical evaluation and most importantly ensuring that the core feature in true mixed methods (as opposed to multi-methods) designs – analytical and methodological integration – is implemented. To be able to conduct mixed methods approaches that are rigorously designed, logically executed, and transparently reported, we need to move to a position where funders, researchers, journal editors and research consumers demand methodological integration of methods and data from study outset, rather than as a mere afterthought in the discussion section of research reports.

## SPEAKER PRESENTATIONS

### S1 Exploring complexity in systematic reviews of complex interventions

#### Sascha Köpke^1^, Jane Noyes^2^, Jackie Chandler^3^

##### ^1^University of Lübeck, Germany; ^2^Bangor University, UK; ^3^Cochrane Collaboration, Oxford, UK

###### **Correspondence:** Gabriele Meyer (Gabriele.Meyer@medizin.uni-halle.de ) – Martin Luther University Halle-Wittenberg, Magdeburger Str. 8, D-06112 Halle (Saale), Germany

There is a lot of discussion about the most adequate approaches to perform systematic reviews and meta-analyses of complex interventions. Reviewers need to broaden their view and to have a detailed look at all available evidence for the whole process of developing and evaluating complex interventions. Decisions about approaches should acknowledge intervention aims. Decisions about the scope and methods of the systematic review depend on the available information, which should be assessed in view of clinicians’ and patients’ needs. Important decisions include the type of evidence to be searched and the choice of methods to describe or synthesise it. This will usually require a combination of different study types including quantitative and qualitative data. Transparent reporting is a key issue to allow readers to apply the findings to specific contexts.

### S2 Can complex health interventions be optimised before moving to a definitive RCT? Strategies and methods currently in use

#### Sara Levati (Sara.Levati@gcu.ac.uk)

##### NMAHP Research Unit, Glasgow Caledonian University, UK

RCTs are recognised as the ‘gold standard’ methodology in the evaluation of complex interventions, however, they often fail to detect whether the lack of intervention effect is due to sub optimal design, implementation failure or genuine ineffectiveness. Given the current financial constraints upon health services research, it is increasingly important to define pre-trial optimisation methods that can give indications on how the intervention works and help maximise chances for the intervention to be effective. This scoping review provides a map of the health literature on pre-trial strategies aimed at optimising complex interventions before a RCT and a snapshot of the methods currently used. Scholarly literature was searched using MEDLINE, CINAHL, AMED, PsycINFO and ProQuest Nursing & Allied Health Source for papers published between January 2000 and March 2015 available in English language. The literature search identified 3940 unique references, 27 of which met the inclusion criteria. Optimisation strategies explored the feasibility and acceptability of the intervention to patients and healthcare professionals, estimated the effectiveness and cost-effectiveness of different combinations of components and identified potential barriers to implementation. Large variations in the methods adopted were noted across studies, including interviews and focus groups with interventions’ providers and receivers, experts consultations, economic modelling, small uncontrolled pilot studies and evaluation questionnaires. Overall, there is the potential for optimisation strategies to detect, in a cost-effective way, those interventions and components that are likely to fail or show little effect if implemented in a full-scale RCT.

### S3 A systematic approach to develop theory based implementation interventions

#### Anne Sales (salesann@med.umich.edu)

##### School of Nursing, University of Michigan, USA and Center for Clinical Management Research, VA Ann Arbor Healthcare System, USA

In this presentation, I will briefly review the large landscape of frameworks and theories in implementation research, and suggest an approach to designing implementation interventions that builds on current widely cited frameworks to develop theory-based implementation interventions.

### S4 Pilot studies and feasibility studies for complex interventions: an introduction

#### Lehana Thabane^1^, Lora Giangregorio^2^

##### ^1^McMaster University, Canada; ^2^University of Waterloo, Canada

###### **Correspondence:** Lehana Thabane (thabanl@mcmaster.ca) – McMaster University, Canada

Pilot studies for phase III trials—which are comparative randomized trials designed to provide preliminary evidence on the clinical efficacy of an intervention—are routinely performed in many clinical areas. Also commonly known as “feasibility”, “dress rehearsal” or “vanguard” studies, they are designed to assess recruitment potential; to assess the feasibility of international collaboration or coordination for multicentre trials; to increase clinical experience with the study intervention for the phase III trials. They are the best way to assess feasibility of a large, expensive full-scale study, and in fact are an almost essential pre-requisite (Thabane et al (*BMC Medical Research Methodology* 2010, 10:1). Conducting a pilot prior to the main study can enhance the likelihood of success of the main study and potentially help to avoid doomed main studies. The presentation will cover some key aspects of pilot studies for phase III trials of complex interventions including: 1) reasons for conducting a pilot study; 2) misconceptions about pilot studies; 3) criteria for evaluating the success of a pilot study; 4) differences and similarities between pilot and feasibility studies; and 5) a brief update on the development of CONSORT extension for pilot trials.

### S5 What can be done to pilot complex interventions?

#### Nancy Feeley^1^, Sylvie Cossette^2^

##### ^1^Ingram School of Nursing, McGill University, and Centre for Nursing Research and Lady Davis Institute, Jewish General Hospital, Montreal, Québec, Canada; ^2^Faculty of Nursing, University of Montreal, and Montreal Heart Institute Research Center, Montreal, Québec, Canada

###### **Correspondence:** Nancy Feeley (nancy.feeley@mcgill.ca) – Ingram School of Nursing, McGill University, and Centre for Nursing Research and Lady Davis Institute, Jewish General Hospital, Montreal, Québec, Canada

In a pilot or feasibility study, the feasibility and acceptability of many of the key features of a complex intervention can be examined. The main research questions are: Can the intervention be provided as planned? Is the intervention acceptable to participants? Certain features (e.g., content, sequence, dose, timing, mode of delivery) will be more important to scrutinize than others, depending on the particular challenges anticipated and the questions that remain at the end of intervention development. This presentation will examine which intervention features might be examined in a pilot study, and describe examples of studies that have done so. In addition, special issues such as contamination and co-intervention will be discussed.

### S6 Using feasibility and pilot trials to test alternative methodologies and methodological procedures prior to full scale trials

#### Rod Taylor (R.Taylor@exeter.ac.uk)

##### University of Exeter Medical School, South Cloisters, St Luke’s Campus, UK

Feasibility and pilot studies play a key role in health research, in providing information for the planning of full scale randomised controlled trials. Medical Research Council guidance for development and evaluation of complex interventions states that pilot and feasibility studies are essential in the development and testing of an intervention prior to a large-scale evaluation. Before committing investment in costly and time-consuming full scale trials, funding bodies increasingly demand that investigators provide evidence from pilot/feasibility studies addressing the question: “Can this full scale trial be done?” This presentation will: (i) identify the common methodological uncertainties in the design and execution of full scale trials; (ii) review approaches to testing these methodological uncertainties in feasibility/pilot studies; and (iii) discuss the design and selection of feasibility and pilot studies to best address these uncertainties. These concepts will be illustrated by reference to recent feasibility/pilot studies.

### S7 A mixed methods feasibility study in practice

#### Jacqueline Hill^1^, David A Richards^2^, Willem Kuyken^3^

##### ^1^Mood Disorders Centre, School of Psychology, University of Exeter, UK; ^2^University of Exeter Medical School, UK; ^3^Department of Psychiatry, University of Oxford, UK

###### **Correspondence:** Jacqueline Hill (j.j.hill@exeter.ac.uk) – Mood Disorders Centre, School of Psychology, University of Exeter, UK

Depression is set to become the third biggest cause of the global burden of disease by 2030 yet access to treatment is poor. To maximise access, worldwide psychological therapies for depression are delivered using a system called stepped care. However, we do not know if this system achieves similar patient benefit for less cost compared with alternatives. A fully-powered clinical trial of stepped care is required but currently prevented by a number of uncertainties. This presentation will describe how we chose to answer these in a single feasibility study encompassing a pilot randomised controlled trial and embedded qualitative interviews. In particular, the presentation will focus on the innovative use of integrated mixed methods analysis to explore how patients’ views of stepped care and the number of treatment sessions they attend relate. In this way, we will illustrate how it is possible to interweave quantitative and qualitative data in original ways as part of a feasibility study to address multiple clinical, procedural and methodological uncertainties.

### S8 Non-standard experimental designs and preference designs

#### Louise von Essen (louise-von.essen@pubcare.uu.se)

##### Department of Public Health and Caring Sciences, Uppsala University, Sweden

Randomized controlled trials (RCT) have been accepted as the ‘gold standard’ design for evaluating the effectiveness of a single intervention such as a drug. However, recruitment to clinical trials may be affected by the choice of intervention that participants might make, if they were allowed to choose, and by whether they actually receive their preferred intervention. These effects cannot be estimated in the RCT design. Additionally the RCT runs counter to the current emphasis on patient choice which is the cornerstone in many governments’ current health strategies and where preferences or ethical objections to an RCT exist, alternatives that replicate more closely the behaviors in real world health care rather than conform to standard trial design should be considered. Citizen participation in research is advocated by governments, research councils, and other funding bodies and as the public, patients, and significant others become more involved in research activities they may be unwilling to be passive participants of a research randomization process. Non-standard and preference designs go some way towards addressing the problems of low recruitment and retention rates and non-implementation of results of clinical research. Advantages and challenges e.g. with regard to internal and external validity with non-standard and preference designs such as the comprehensive cohort design, pre-randomized design, cohort multiple randomized controlled trial, and two-stage randomized design will be presented at the conference.

### S9 Evaluation gone wild: using natural experimental approaches to evaluate complex interventions

#### Andrew Williams (A.J.Williams@ed.ac.uk or a.williams2@exeter.ac.uk)

##### Farr Institute, Scotland, and the Scottish Collaboration for Public Health Research and Policy, 20 West Richmond Street (East Wing), Edinburgh EH8 9DX, UK

Any outcome evaluation of an intervention needs to address the following questions:

1. Did change occur?

2. Was the change due to the intervention?

3. Was the change significant/meaningful? (Sanson-Fisher et al, 2014) [1]

With the ability to calculate the unbiased attributable effects on an intervention the randomised controlled trial (RCT) is seen as the ‘gold standard’ method for answering all three questions. However, as complex interventions are recognised as events within systems, they are rarely tame enough for RCTs to be feasible (Hawe et al., 2009) [2]. The natural experimental approaches (NEAs) permit quasi-experimental evaluation which can answer the three questions when the intervention is wilder, particularly when routinely collected outcome data are available (Craig et al., 2011) [3].

Robust NEAs rely on quasi-randomised identification (instrumental variable, regression discontinuity or propensity score matching) of a control (counterfactual) population to compare with the intervention population. Subsequently, taking a difference in differences or controlled before-after approach permits the calculation of an effect attributable to the intervention addressing the three questions. Quasi-randomised scenarios often occur by chance (naturally, not designed as part of an evaluation) within complex interventions allowing valuable prospective or retrospective evaluation. For example, criterion-based intervention eligibility permitting a regression discontinuity design. Although not faultless, NEAs offer an often missed opportunity to evaluate complex interventions.

**References**

1. Sanson-Fisher, R.W., D'Este, C.A., Carey, M.L., Noble, N., Paul, C.L., 2014. Evaluation of systems-oriented public health interventions: alternative research designs. Annual review of public health 35:9-27. doi: 10.1146/annurev-publhealth-032013-182445

2. Hawe, P. Shiell, A., Riley, T., 2009. Theorising interventions as events in systems. American journal of community psychology 43:267-76. doi: 10.1007/s10464-009-9229-9

3. Craig, P., Cooper, C., Gunnell, D., Haw, S., Lawson, K., Macintyre, S., Ogilvie, D., Petticrew, M., Reeves, B. et al., 2011. Using natural experiments to evaluate population health interventions: guidance for producers and users of evidence. Medical Research Council, London. http://www.mrc.ac.uk/documents/pdf/natural-experiments-guidance/ (accessed 03 December 2014).

### S10 The stepped wedge cluster randomised trial: an opportunity to increase the quality of evaluations of service delivery and public policy interventions

#### Karla Hemming^1^, Richard Lilford^2^, Alan Girling^3^, Monica Taljaard^4^

##### ^1^University of Birmingham Medical School, UK; ^2^Warwick Medical School, University of Warwick, Coventry, UK; ^3^Institute of Applied Health Research, College of Medical and Dental Sciences, University of Birmingham, UK; ^4^Clinical Epidemiology Program, Ottawa Hospital Research Institute, Ottawa, Canada

###### **Correspondence:** Karla Hemming (K.HEMMING@bham.ac.uk) – University of Birmingham Medical School, UK

The stepped wedge cluster randomised trial (SW-CRT) is a novel research study design that is increasingly being used in the evaluation of service delivery type interventions. The design involves random and sequential crossover of clusters from control to intervention, until all clusters are exposed. We illustrate the use of the design by giving case examples, summarise the results of an update of a methodological systematic review of the quality of reporting and provide recommendations for reporting and analysis. The use of the SW-CRT is rapidly increasing and areas of use are diverse. We illustrate how the design is being used to evaluate the effectiveness of a complex intervention, being rolled-out across 90 UK hospitals, to reduce mortality in patients undergoing emergency laparotomy. Quality of reporting is found to be low. In a SW-CRT more clusters are exposed to the intervention towards the end of the study than in its early stages. A result which *prima facia* might look to be suggestive of an effect of the intervention may therefore transpire to be the result of a positive underlying temporal trend. A large number of studies do not report how they allowed for temporal trends in the design or analysis. The SW-CRT is a pragmatic study design which can reconcile the need for robust evaluations with political or logistical constraints. Quality and reporting is generally low and so consensus guidelines on reporting and analysis are urgently needed.

### S11 Adaptive designs in confirmatory clinical trials: opportunities in investigating complex interventions

#### Munyaradzi Dimairo (m.dimairo@sheffield.ac.uk)

##### Clinical Trials Research Unit, University of Sheffield, UK

Adaptive designs are now considered innovative alternative trial designs with potential to improve efficiency in clinical trials research when appropriately used to answer research questions. Their appropriate implementation in confirmatory trials is gaining widespread attention among researchers, public funders and regulators. Based upon findings from our review of confirmatory adaptive designs, this talk focuses on acceptable scope with huge potential to be implemented in confirmatory trials setting. In theory, although adaptive designs could be applied across a wide range of study interventions, attention will be given to complex interventions, with relevant examples without focusing much on technical statistical details. This study is funded by the NIHR investigating the use of adaptive designs in publicly funded confirmatory trials with an aim to come up with a guidance document and recommendations for their appropriate use.

### S12 Processes, contexts and outcomes in complex interventions, and the implications for evaluation

#### Mark Petticrew (Mark.Petticrew@lshtm.ac.uk)

##### Faculty of Public Health and Policy, London School of Hygiene & Tropical Medicine, UK

It would be convenient if there were two categories of interventions - simple and complex – but of course this is not the case. Instead interventions can be placed on a spectrum of complexity. For simpler interventions there is often a relatively well-understood (or perhaps just well-accepted) causal pathway between the intervention and its outcomes. This may derive from basic science - for example if the physiological pathways between particular medical interventions and changes in outcomes have been previously shown. For more complex interventions - those in which the intervention interacts with and adapts within its social context - such pathways may be less well understood, less predictable (or even chaotic) and, crucially, non-linear. Here, the intervention may not even be easily distinguishable from the context. In such situations it may be more productive to move away from identifying “interventions” and “components,” towards seeing interventions as changes systems (Hawe et, al. 2009) [1] rather than as discrete events, or as “packages” of interconnected elements. Such a perspective has major implications for how we conceptualise and evaluate public health interventions. This presentation will discuss the implications for evaluation, using recent examples from public health.

**References**

1. Hawe, P. Shiell, A., Riley, T., 2009. Theorising interventions as events in systems. Am J Community Psychol 43:267-276. doi: 10.1007/s10464-009-9229-9

### S13 Processes, contexts and outcomes in complex interventions, and the implications for evaluation

#### Janis Baird^1^, Graham Moore^2^

##### ^1^MRC Lifecourse Epidemiology Unit, University of Southampton, UK; ^2^DECIPHer, Cardiff University, UK

###### **Correspondence:** Janis Baird (jb@mrc.soton.ac.uk) – MRC Lifecourse Epidemiology Unit, University of Southampton, UK

Attempts to address health problems increasingly involve the development and evaluation of ‘complex interventions’. In addition to outcomes evaluation, understanding complex intervention mechanisms, how interventions are implemented, and how they produce change in specific contexts, is essential if findings are to inform policy and practice. For this, process evaluation is vital. MRC guidance for evaluating complex interventions, published in 2008, recognised the value of process evaluation alongside trials of effectiveness, though provided little insight into how to conduct process evaluation. In 2010, a workshop funded by the MRC Population Health Science Research Network (PHSRN) identified a need for guidance to fill this gap. Following the workshop, 11 researchers with experience of public health evaluations developed draft guidance, drawing upon reviews of the literature, group discussions of case studies and feedback obtained at a number of conference workshops. Academic, policy and practice stakeholders were also consulted during the development of the guidance. The new guidance sets out a framework for linking core functions of process evaluation, understanding implementation, mechanisms of impact and context. It provides assistance in planning, designing, conducting, reporting and appraising process evaluations, and argues for a systematic approach to designing and conducting process evaluations, drawing on clear descriptions of intervention theory and identification of key empirical uncertainties. While acknowledging that each process evaluation will be different, the guidance assists in thinking through common decisions to be made when planning and conducting a process evaluation, and provides a framework for peer review.

### S14 Qualitative evaluation alongside RCTs: what to consider to get relevant and valuable results

#### Willem Odendaal^1^, Salla Atkins^2^, Elizabeth Lutge^3^, Natalie Leon^4^, Simon Lewin^1,5^

##### ^1^Health Systems Research Unit, South African Medical Research Council, South Africa (http://www.mrc.ac.za/healthsystems/healthsystems.htm); ^2^Karolinska Institutet, Stockholm, Sweden; ^3^Epidemiology, Health Research and Knowledge Management, KwaZulu-Natal Department of Health, South Africa; ^4^South African Medical Research Council, South Africa; ^5^Global Health Unit, Norwegian Knowledge Centre for the Health Services, Norway

###### **Correspondence:** Willem Odendaal (Willem.Odendaal@mrc.ac.za) – Health Systems Research Unit, South African Medical Research Council, South Africa (http://www.mrc.ac.za/healthsystems/healthsystems.htm)

Qualitative evaluations are becoming increasingly commonplace alongside RCTs. They provide useful insight into the way things work within RCTs, particularly insight into the how and why things work, and contextualise RCT effects. While there are established guidelines for conducting qualitative research, there is less guidance on how to go about qualitative process evaluations alongside RCTs. Our presentation will discuss how to enhance the utility of qualitative evaluations in explaining RCT effects, through identifying key issues, such as involvement of trialists in the evaluation; scaling the evaluation; and maintaining the integrity of the trial. We discuss these issues by reflecting on practical examples of qualitative evaluations conducted in South Africa.

### S15 Using economic evaluations to understand the value of complex interventions: when maximising health status is not sufficient

#### Katherine Payne (Katherine.Payne@manchester.ac.uk)

##### The University of Manchester, UK

The economic evaluation of complex interventions may be problematic for many reasons generally underpinned by ‘complexity’ of the intervention but more specifically because of the potential number of relevant patient outcomes. This presentation will begin by presenting an overview of methods of economic evaluation used by decision-making bodies such as the National Institute for Health and Care Excellence and then describe a programme of work that focused on clinical genetics services, as an example of a complex intervention, to illustrate why sometimes maximising health status alone is not sufficient. Genetic services and tests, are a good example of a complex intervention and have broader objectives than just health gain, which may usefully be measured using the concept of capability to make an informed decision. The presentation will conclude by describing the further methodological work required to ensure the methods to value outcomes continue to consider opportunity cost and are therefore, suitable for informing resource allocation decisions with the constraints of a finite healthcare budget.

### S16 How to arrive at an implementation plan

#### Theo van Achterberg (theo.vanachterberg@med.kuleuven.be)

##### Leuven University KU Leuven, Belgium

The presentation “How to arrive at an implementation plan” will highlight the most important steps to be taken in the development of an implementation plan, using examples from research and practice. These include 1) analysing barriers and facilitators for implementation and linking these to relevant concepts, 2) using these concepts to find and consider theories that can inform the selection of implementation strategies, 3) considering effectiveness of common strategies, and 4) collaborating with target groups and stakeholders in operationalising strategies into a practical implementation plan. Well considered preparations such as these are crucial to implementation success, and thus optimising chances of effective improvement in healthcare.

### S17 Modelling process and outcomes in complex interventions

#### Walter Sermeus (walter.sermeus@telenet.be)

##### School of Public Health & Primary Care, Leuven University KU Leuven, Belgium

In this presentation, I focus on probably the least studied and understood element of developing a complex intervention: modelling the complex intervention by putting together all ‘active components’ that are known to have an effect based on empirical evidence or theory. I will discuss several problems: What active ingredients to select? How do we know that the intervention is the best one? How to optimise? How to anticipate on barriers in implementation? These problems will be discussed making use of a practical example.

### S18 Systems modelling for improving health care

#### Martin Pitt^1^, Thomas Monks^2^

##### ^1^University of Exeter Medical School, UK; ^2^Faculty of Health Sciences, University of Southampton, UK

###### **Correspondence:** Martin Pitt (M.Pitt@exeter.ac.uk) – University of Exeter Medical School, UK

The growing complexity of health care coupled with the ever-increasing pressures to ensure efficient and effective use of limited resources have encouraged policy makers to turn to system modelling solutions. Such techniques have been available for decades, but despite ample research which demonstrates potential, their application in health services to date is limited. This presentation surveys the breadth of approaches available to support delivery and design across many areas and levels of healthcare planning. Case studies will be presented to exemplify how impactful application of health system modelling can be achieved and demonstrate how policy making can benefit from the application of these tools. This is followed by a discussion of the key issues surrounding the use of these methods in health, what barriers need to be overcome to ensure more effective implementation, and the likely developments in the future.

