# Erratum to: Researching Complex Interventions in Health: The State of the Art

**DOI:** 10.1186/s12913-016-1416-4

**Published:** 2016-05-13

**Authors:** Peter Craig, Ingalill Rahm-Hallberg, Nicky Britten, Gunilla Borglin, Gabriele Meyer, Sascha Köpke, Jane Noyes, Jackie Chandler, Sara Levati, Anne Sales, Lehana Thabane, Lora Giangregorio, Nancy Feeley, Sylvie Cossette, Rod Taylor, Jacqueline Hill, David A. Richards, Willem Kuyken, Louise von Essen, Andrew Williams, Karla Hemming, Richard Lilford, Alan Girling, Monica Taljaard, Munyaradzi Dimairo, Mark Petticrew, Janis Baird, Graham Moore, Willem Odendaal, Salla Atkins, Elizabeth Lutge, Natalie Leon, Simon Lewin, Katherine Payne, Theo vanAchterberg, Walter Sermeus, Martin Pitt, Thomas Monks

**Affiliations:** MRC/CSO Social and Public Health Sciences Unit (SPHSU), University of Glasgow, 200 Renfield Street, Glasgow, G2 3QB UK; Lund University, PO Box 117, 221 00, Lund, G2 3QB Sweden; University of Exeter Medical School, South Cloisters, St Luke’s Campus, Exeter, EX1 2LU UK; Department of Caring Science, Malmö University, SE-205 06 Malmö, Sweden; Martin Luther University Halle-Wittenberg, Magdeburger Str. 8, D-06112 Halle (Saale), Germany; University of Lübeck, Lübeck, Germany; Bangor University, Bangor, UK; Cochrane Collaboration, Oxford, UK; NMAHP Research Unit, Glasgow Caledonian University, Glasgow, UK; School of Nursing, University of Michigan, USA and Center for Clinical Management Research, VA Ann Arbor Healthcare System, Michigan, USA; McMaster University, Hamilton, Canada; University of Waterloo, Waterloo, Canada; Ingram School of Nursing, McGill University, and Centre for Nursing Research and Lady Davis Institute, Jewish General Hospital, Montreal, Québec Canada; Faculty of Nursing, University of Montreal, and Montreal Heart Institute Research Center, Montreal, Québec Canada; University of Exeter Medical School, South Cloisters, St Luke’s Campus, Exeter, UK; Mood Disorders Centre, School of Psychology, University of Exeter, Exeter, UK; University of Exeter Medical School, Exeter, UK; Department of Psychiatry, University of Oxford, Oxford, UK; Department of Public Health and Caring Sciences, Uppsala University, Uppsala, Sweden; Farr Institute, Scotland, and the Scottish Collaboration for Public Health Research and Policy, 20 West Richmond Street (East Wing), Edinburgh, EH8 9DX UK; University of Birmingham Medical School, Birmingham, UK; Warwick Medical School, University of Warwick, Coventry, UK; Institute of Applied Health Research, College of Medical and Dental Sciences, University of Birmingham, Birmingham, UK; Clinical Epidemiology Program, Ottawa Hospital Research Institute, Ottawa, Canada; Clinical Trials Research Unit, University of Sheffield, Sheffield, UK; Faculty of Public Health and Policy, London School of Hygiene & Tropical Medicine, London, UK; MRC Lifecourse Epidemiology Unit, University of Southampton, Southampton, UK; DECIPHer, Cardiff University, Cardiff, UK; Health Systems Research Unit, South African Medical Research Council, Tygerberg, South Africa; Karolinska Institutet, Stockholm, Sweden; Epidemiology, Health Research and Knowledge Management, KwaZulu-Natal Department of Health, Durban, South Africa; South African Medical Research Council, Tygerberg, South Africa; Global Health Unit, Norwegian Knowledge Centre for the Health Services, Oslo, Norway; The University of Manchester, Manchester, UK; Leuven University KU Leuven, Leuven, Belgium; School of Public Health & Primary Care, Leuven University KU Leuven, Leuven, Belgium; University of Exeter Medical School, Exeter, UK; Faculty of Health Sciences, University of Southampton, Southampton, UK

Unfortunately, the original version of this article [1] contained an error in abstract S1. The corresponding author for the abstract ‘Gabriele Meyer’ was included in the 'Correspondence' section but accidentally omitted from the author list. The correct author list should be as below:

Gabriele Meyer^1*^, Sascha Köpke^2^, Jane Noyes^3^, Jackie Chandler^4^

^1^Martin Luther University Halle-Wittenberg, Magdeburger Str. 8, D-06112 Halle (Saale), Germany

^2^University of Lübeck, Germany

^3^Bangor University, UK

^4^Cochrane Collaboration, Oxford, UK

Correspondence: Gabriele Meyer (Gabriele.Meyer@medizin.uni-halle.de) – Martin Luther University Halle-Wittenberg, Magdeburger Str. 8, D-06112 Halle (Saale), Germany
